# The Optimization of the One-Pot Synthesis of Au@SiO_2_ *Core–Shell* Nanostructures: Modification with Dansyl Group and Their Fluorescent Properties

**DOI:** 10.3390/ma17102213

**Published:** 2024-05-08

**Authors:** Agata Kowalska, Elżbieta Adamska, Anna Synak, Beata Grobelna

**Affiliations:** 1 Faculty of Chemistry, University of Gdansk, Wita Stwosza 63, 80-308 Gdansk, Poland; agata.kowalska@phdstud.ug.edu.pl (A.K.); elzbieta.adamska@ug.edu.pl (E.A.); 2 Faculty of Mathematics, Physics and Informatics, University of Gdansk, Wita Stwosza 57, 80-308 Gdansk, Poland; anna.synak@ug.edu.pl

**Keywords:** *core–shell* nanostructures, gold nanoparticles, silica coating, nanomaterials, fluorescence

## Abstract

This work describes the optimization of the one-pot synthesis of fine *core–shell* nanostructures based on nanogold (Au NPs) and silica (SiO_2_). The obtained *core–shell* nanomaterials were characterized by Transmission Electron Microscopy (TEM and by the method of spectroscopes such as UV–Vis Spectroscopy and Fourier Transform Infrared Spectroscopy (FT-IR). In addition, the measurement of the zeta potential and size of the obtained particles helped present a full characterization of Au@SiO_2_ nanostructures. The results show that the influence of reagents acting as reducers, stabilizers, or precursors of the silica shell affects the morphology of the obtained material. By controlling the effect of the added silica precursor, the thickness of the shell can be manipulated, the reducer has an effect on the shape and variety, and then the stabilizer affects their agglomeration. This work provides also a new approach for Au@SiO_2_ *core–shell* nanostructure preparation by further modification with dansyl chloride (DNS–Cl). The results show that, by tuning the silica shell thickness, the intensity of the fluorescence spectrum of Au@SiO_2_–(CH_2_)_3_–NH–DNS nanocomposite is about 12 times higher than that of DNS–Cl.

## 1. Introduction

In the last decades, gold nanoparticles have been of great interest; they differ from gold on a macro scale by their optical properties. These optical features primarily lead to their use as an element in the construction of sensors, which can be used in many fields, including industrial, medical, biological, or food analysis. The wide range of potential applications of nanomaterials based on Au NPs, are prompting researchers to turn more often to these types of material. Moreover, their functionality can be confirmed by using various methods such as fluorescence, Surface Plasmon Resonance (SPR) [1], Surface Enhanced Raman Scattering (SERS) [2], Plasmon Enhanced Fluorescence (PEF), and Metal Enhanced Fluorescence (MEF) [3]. The widest areas of application of Au NPs are medicine and the cosmetics industry [4,5,6,7,8]. In medicine, one of the most important applications is the diagnosis and treatment of cancer, where nanogold is used in such imaging techniques as computed tomography, fluorescence imaging, photoacoustic imaging, magnetic resonance imaging, and others [4,5]. On the other hand, in the cosmetics industry, Au NPs have gained favor due to their regenerative properties, supporting the synthesis of collagen in the skin. Formulations containing Au NPs have an anti-glycation effect while repairing damaged collagen [6] or reducing the number of free radicals [7]. In addition, due to their high stability, they can act as a carrier of active substances in cosmetic formulations, by which the substance will reach the deeper layers of the skin [8].

Despite having many advantages, gold nanoparticles may be toxic, and their tendency to form larger agglomerates may weaken their optical properties [9]. Therefore, more and more attention is paid to the combination of nanoparticles with neutral materials such as silica, whose task is to protect the core against physical and chemical factors, ensuring colloidal stability, degradability, good dispersity, and the possibility of further modification of the nanomaterial [10,11]. The *core–shell* structures created in this way are multifunctional [12] and differ from other particles by their unique composition and combined properties of the core and shell [13]. Therefore, in the last decade, a wide range of *core–shell* materials have been developed for various applications [11,14]. The surface of the shell can be modified and then functionalized with fluorescence molecules. Because of this, functionalized *core–shell* nanostructures have been selected as a proper material for application in the design of new plasmonic platforms [3,15,16]. Furthermore, the shell offers chemical inertness and robustness, and it ensures the occurrence of the MEF phenomenon, whose efficiency is strongly dependent on the distance between the metal and fluorophore [17]. The effect of fluorescence quenching occurs when the distance between gold and fluorophore is less than 4 nm [18,19].

This work reports on the one-step synthesis of a direct nanocomposite from a gold core surrounded by a silica coating and the optimization of this process by changing the synthesis environment by manipulating the amounts of added substrates. To the best of our knowledge, this is the first study on such a procedure for obtaining Au@SiO_2_ with hydrazine as a reducing agent and showing the entire optimization of the nanostructure preparation process. It especially shows the influence of reagents on the morphology of the obtained nanostructures. Moreover, using the method developed by our group, the number of depositions of amino groups (–NH_2_) on the surface of nanostructures was also calculated. In addition, to broaden the scope of application, the surface was modified and functionalized with dansyl chloride (DNS–Cl) to obtain a new plasmonic platform, which can be potentially used in analyte detection, thin-film solar cells, targeted therapies as drug carriers, and biosensors [20,21].

## 2. Materials and Methods

### 2.1. Materials and Reagents

All reagents and solvents were of analytical grade and were used without purification. Tetrachloroauric acid (HAuCl_4_), sodium citrate dihydrate (C_6_H_5_Na_3_O_7_·2H_2_O), ethanol (C_2_H_5_OH), aqueous ammonia (NH_3·_H_2_O; 25%), tetraethyl orthosilicate (SiC_8_H_20_O_4_, TEOS), hydrazine monohydrate (N_2_H_4_·H_2_O; 40%), hexadecyltrimethylammonium bromide (C_19_H_42_BrN, CTAB), toluene (C_7_H_8_), (aminopropyl)trimethoxysilane (H_2_N(CH_2_)_3_Si(OCH_3_)_3_, APTMS), Fmoc–glycine (C_17_H_15_NO_4_, Fmoc–Gly–OH), N,N′-diisopropylcarbodiimide (C_7_H_14_N_2_, DIC), N,N-dimetyloformamid (C_3_H_7_NO, DMF), methylene chloride (CH_2_Cl_2_), and dansyl chloride (DNS-Cl) reagents were purchased from Sigma-Aldrich (Poznan, Poland). All the samples were prepared using deionized water produced by the Hydrolab system installed in our laboratory.

### 2.2. Synthesis of Nanoparticles and Nanocomposites

#### 2.2.1. Synthesis of Au NPs

The Turkevich method was used to obtain gold nanoparticles [22]. For this purpose, 0.005 g of tetrachloroauric acid precursor was dissolved in 50 mL of water. Then, 0.3 mL of a 50 mg/mL sodium citrate solution was prepared, brought to a boil, and added to the previously prepared solution. In the next stage, the solution was stirred with a magnetic stirrer and heated until a dark red color appeared.

#### 2.2.2. Synthesis of SiO_2_ NPs

Silica nanoparticles were obtained using the Stöber sol–gel method [23]. For this purpose, a silica sol was prepared by mixing 95 mL of ethanol, 3.60 mL of deionized water, 3 mL of 25% aqueous ammonia, and 10 mL of TEOS. The whole mixture was stirred on a magnetic stirrer for about 30 min. The last step was to centrifuge the sol and wash the precipitate several times with water and ethanol.

#### 2.2.3. Synthesis of Au@SiO_2_ *Core–Shell* Nanostructures

The one-step method was used to synthesize a nanocomposite consisting of a gold core surrounded by a silica shell. The reducing agent (hydrazine) was mixed with 400 mL of an aqueous solution that contained a surfactant (CTAB). This mixture was then magnetically stirred at room temperature for about 2 min. Subsequently, a 10 mL aqueous solution of tetrachloroauric acid with a concentration of 7.35 × 10^−2^ M was prepared. The solution thus obtained was slowly added dropwise to the previously prepared mixture containing hydrazine and CTAB and was stirred. While stirring, 100 mL of ethanol and 4 mL of 25% aqueous ammonia solution were added. At the very end, TEOS, a silica precursor reagent, was added, and the reactions were carried out by stirring for 2 h. The last step was centrifugation of the obtained sludge and cleaning by washing it several times with water and ethanol.

### 2.3. Surface Modification and Fluorescent Properties

#### 2.3.1. Surface Modification with Aminopropyl Groups (–(CH_2_)_3_–NH_2_)

The next steps to modify the surface of the obtained nanostructures were based on the procedures previously described by our team [15]. Attachment of aminopropyl groups to nanostructures consisted of weighing 70 mg of the previously obtained Au@SiO_2_ sample; then, 6 mL of toluene was added and left for 15 min in an ultrasonic bath. Then, 2 mL of APTMS was added, stirred on a magnetic stirrer, and heated in an oil bath at 120 °C for 24 h. After this time, the sample was centrifuged and purified with methylene chloride.

#### 2.3.2. Synthesis of a Modified Nanocomposite with Fmoc–Gly–OH

To calculate the deposition number, nanostructures with an attached amine group were further modified. Therefore, approximately 10 mg of Au@SiO_2_–(CH_2_)_3_–NH_2_ was mixed with 15 mg of Fmoc–Gly–OH, 50 μL of DIC, 1.5 mL of methylene chloride, and 1.5 mL of DMF. The resulting solution was placed in an ice bath and stirred on a magnetic stirrer for 24 h. The obtained product was centrifuged and washed, first with DMF and methanol, and, finally, with only methanol.

#### 2.3.3. Attachment of DNS–Cl to Modified Au@SiO_2_ Nanostructures

For DNS-Cl attachment, 10 mg of Au@SiO_2_–(CH_2_)_3_–NH_2_ sample and 13 mg of DNS–Cl were weighed and mixed; then, 250 µL of toluene and 3.4 µL of triethanolamine in 900 µL of toluene were added. The solution prepared in this way was stirred on a magnetic stirrer at 80 °C overnight. The last step was to spin the sample and wash it several times with methylene chloride. For further analysis, the centrifuged and washed sample was dispersed in 5 mL of methanol. We also prepared a solution of DNS–Cl in methanol with the same concentration as in the above sample for reference.

### 2.4. Methods

Transmission Electron Microscopy (TEM) measurements were performed using a Tecnai G2 Spirit BioTWIN FEI microscope (Eindhoven, The Netherlands), operated at 120 kV. The samples were dispersed in ethanol and sonicated.

Fourier transform infrared (FT-IR) spectra were taken with a Bruker IFS66 FT-IR spectrometer (Ettlingen, Germany), using the KBr pellet method. Each FT-IR spectrum was recorded between 5000 and 400 cm^−1^.

The zeta potential was measured by Electrophoretic Light Scattering (ELS), with a measurement range of > ±1000 mV, while the particle size measurement was measured via Dynamic Light Scattering (DLS), with a measurement range of 0.3 nm to 10 μm (particle diameter). Both measurements were performed using the Litesizer 500, ANTON-PAAR (Graz, Austria).

Spectrophotometric measurements were made using a UV–Vis spectrophotometer, Perkin Elmer, model Lambda 650 (Shelton, CT, USA). Measurements were made at a temperature of 298 K, using spectrophotometric cuvettes in an aqueous solution. The UV–Vis spectrum was made in the 350–800 nm range.

Steady-state fluorescence measurements were performed using a Carry Eclipse spectrofluorometer (Varian Inc., Melbourne, Australia).

## 3. Results

### 3.1. Synthesis of Nanoparticles and Nanocomposites

This work presents the one-pot method for obtaining nanocomposites consisting of a gold core and a silica shell. In addition, it shows the influence of the manipulation of the added amounts of individual reagents on the morphology of the obtained *core–shell* structures. Gold and silica nanoparticles were also synthesized as references for basic characterization.

Gold nanoparticles were obtained by chemical reduction. Tetrachloroauric acid was used as a precursor, while sodium citrate acted as a reducer and stabilizer. A typical procedure for obtaining silica nanoparticles uses a precursor, which, in this case, was tetraethoxysilane, which was slowly added dropwise to an alcoholic solution containing ammonia water, which acts as a catalyst in this process. The silica nanoparticles obtained in this way were made of amorphous silica.

**Figure 1:**
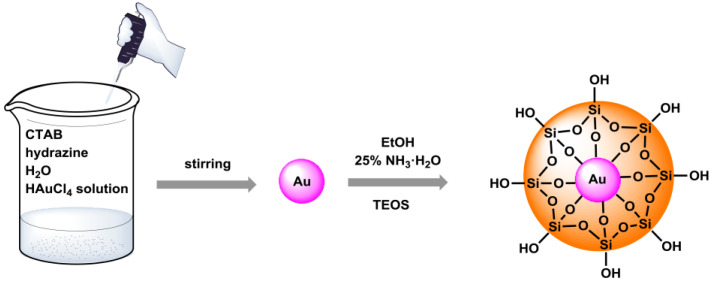
The synthesis route of the presented Au@SiO_2_ nanostructures.

The first step in the synthesis of Au@SiO_2_ *core–shell* nanostructures was to make an aqueous solution containing a surfactant and hydrazine. A solution of tetrachloroauric acid was then added. The last step was the addition of ethanol, ammonia water, and tetraethoxysilane to form a silica shell. The coating of the gold core with silica was carried out using the Stöber method. This process consists of hydrolysis and condensation reactions of tetraethyl orthosilicate, occurring in an alcoholic solution that contains ammonia water, which acts as a catalyst in this process. The schematic course of the performed synthesis is shown in Figure 1. To modify the surface of the nanostructures with amino groups, 3-aminopropyltrimethoxysilane (APTMS) was used in refluxing toluene for 24 h. The procedure was adopted from earlier studies described previously to obtain the *core–shell* nanomaterials [24]. The sample prepared in this way was subjected to further functionalization by covalent attachment of the fluorophore, which is DNS–Cl. We also calculated the deposition number; such studies provide information about how many active amino groups are present in the nanomaterial. Therefore, the method developed by Szczepańska et al. was used to determine the number of Fmoc depositions on the amino groups on Au@SiO_2_ nanostructures. This procedure involves the determination of amino acids anchored on resins that are protected by a fluorenylmethoxycarbonyl (Fmoc) group [25].

**Figure 2:**
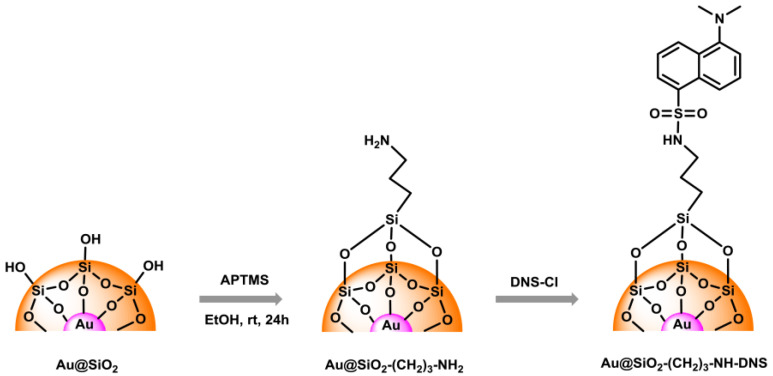
Schematic route to modification and functionalization of Au@SiO_2_ nanostructure.

The obtained samples of Au@SiO_2_ (before and after modification) and SiO_2_ NPs were in the form of powder. Nanosilica was white, while Au@SiO_2_ was dark brown. Nanogold was obtained in the form of a dark red colloidal solution. The diagram of surface modification of nanostructures and subsequent functionalization is shown in Figure 2.

### 3.2. Characterization of Obtained Samples

In order to investigate the influence of selected substrates on the morphology of Au@SiO_2_ nanocomposites, six syntheses with different amounts of reagents were performed. Table 1 contains information about the influence of chosen reagents and their concentration on the morphology of the obtained nanostructures.

**Figure 3:**
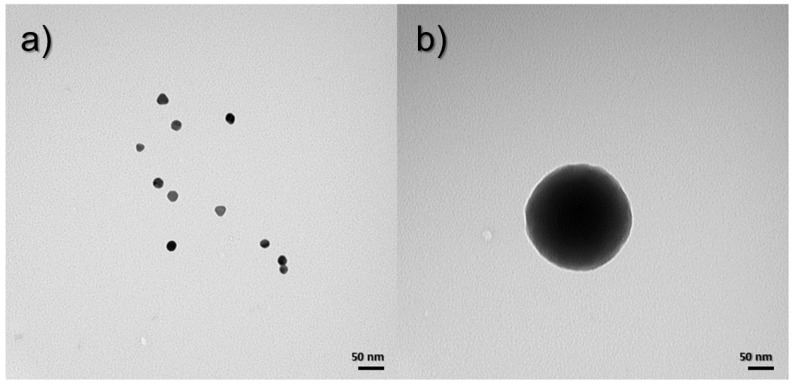
TEM images for (**a**) Au NPs and (**b**) SiO_2_ NPs.

TEM images were taken to determine the morphology of the obtained nanoparticles and nanocomposites. Figure 3a shows an image taken of gold nanoparticles, which have a uniform, spherical shape, and the size oscillates between 20 and 25 nm. On the other hand, Figure 3b shows a silica nanoparticle, whose shape is spherical, and the size varies up to 250 nm.

One of the simplest methods for characterizing the obtained nanomaterials is UV–Vis Spectroscopy. It allows the observation of the maximum absorption, by which the composition of the preparation can be confirmed.

**Figure 4:**
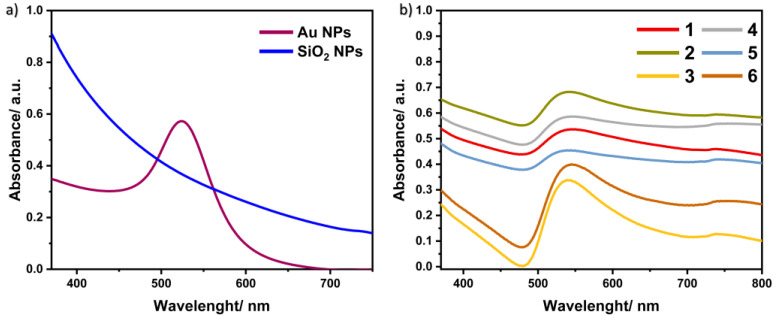
Absorption spectra for (**a**) Au NPs and SiO_2_ NPs and (**b**) all samples of Au@SiO_2_ nanocomposites.

Figure 4 shows the absorption spectra for all synthesized samples. The spectra for Au NPs and SiO_2_ NPs are included in Figure 4a. In the case of gold nanoparticles, the absorption maximum is observed at a wavelength of 530 nm [26]. The same figure also shows the spectrum of silica nanoparticles, for which the maximum absorption in this range was not observed [27]. For the materials, in which the surface of gold nanoparticles is coated with a silica shell, the absorption spectra (Figure 4b) show an absorption maximum in the range of 540–550 nm, which confirms the presence of gold nanoparticles in the samples. Moreover, this range is shifted towards longer wavelengths, which is attributed to the effect of the shell consisting of silica nanoparticles on the structure [28].

**Figure 5:**
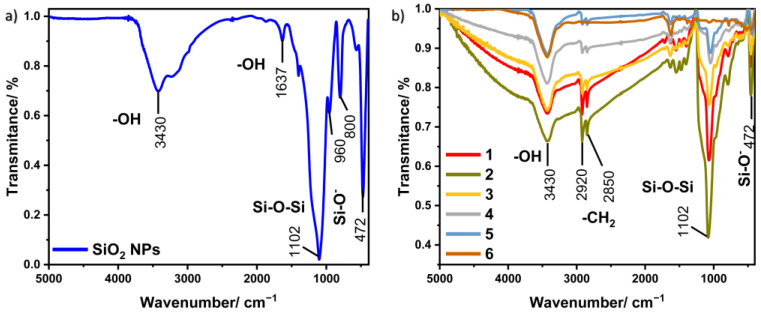
FT-IR spectra for (**a**) SiO_2_ NPs and (**b**) all samples of Au@SiO_2_.

Fourier transform infrared spectroscopy is an analytical technique used to study the molecular structures of the obtained materials. The obtained spectra allow the identification of individual functional groups and, thus, the analysis of the chemical compound. Furthermore, this method allows the detection of functional groups located on the surface of a given material. Figure 5a shows the FT-IR spectrum for silica nanoparticles, which contain several characteristic bands. The band with the maximum located at 3430 cm^−1^ corresponds to the stretching vibrations of the –OH groups, while the maximum at 1637 cm^−1^ is attributed to the bending vibrations of the –OH groups. Signals originating from hydroxyl groups appear in the spectrum due to the presence of water on the surface of the silica. The bands located at 1102, 800, and 472 cm^−1^ can be assigned to asymmetric stretching, symmetrical stretching, and bending vibrations, which are characteristic of the Si–O–Si group. There is also a band at 960 cm^−1^, which is caused by stretching vibrations belonging to the Si–O– groups [29].

FT-IR spectra registered for nine obtained nanocomposites are shown in Figure 5b. All samples have characteristic peaks located at similar wavelengths. In addition to the bands that coincide with those belonging to silica nanoparticles, there are visible bands located in the area around 2920 and 2850 cm^−1^, which can be assigned to asymmetric and symmetrical stretching vibrations, respectively, appearing in the –CH_2_ groups from CTAB [30].

In the case of using *core–shell* materials in medical applications, the important parameters that provide a lot of information are the zeta potential and particle size. The value of the zeta potential depends on many aspects of the composition of the solution or the concentration of ions and surface active particles. In addition, the zeta potential can be used to measure the stability of a colloidal system [31]. In addition, the particle size may be affected by the amounts of reagents used during the preparation of the materials. Both parameters are quite important considering the later applications of nanostructures in the fields of medicine and cosmetology.

**Figure 6:**
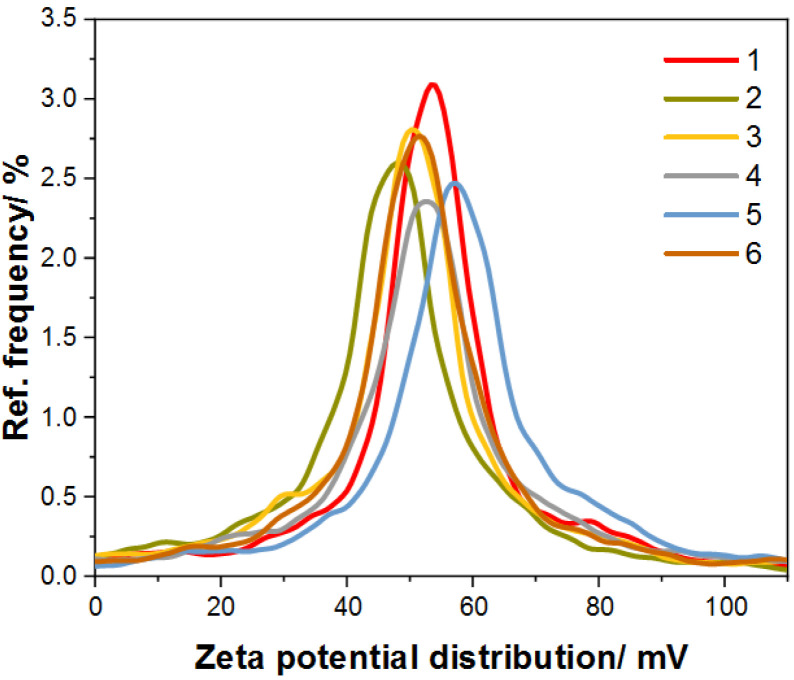
Graph for zeta potential distribution of the synthesized samples of Au@SiO_2_.

It is known that a suspended colloidal system is stable when the zeta potential values are greater than +25 mV or less than −25 mV [32]. Figure 6 shows zeta potential distribution graphs for all obtained Au@SiO_2_ samples; it can be seen that they are characterized by a zeta potential greater than +25 mV, resulting from the use of CTAB as a stabilizer, which has a positive charge. In addition, the detailed results of zeta potential measurement are presented in Table 2 [33].

**Figure 7:**
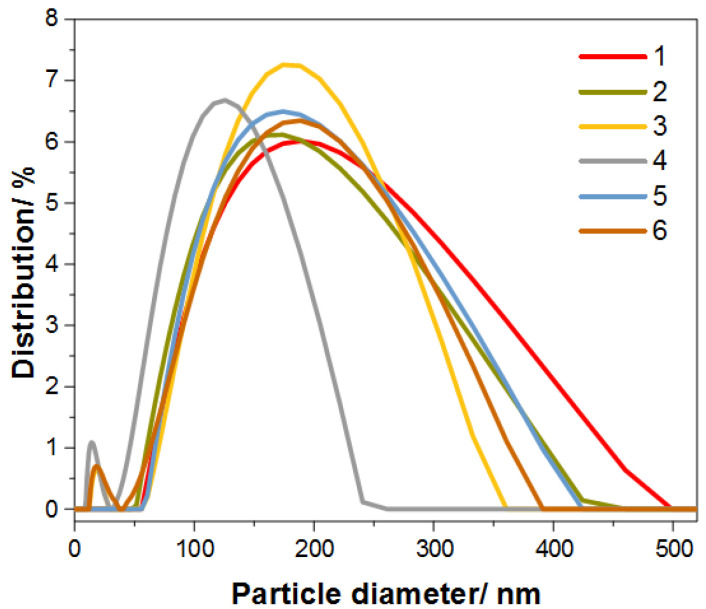
Graph for particle diameter of the synthesized samples of Au@SiO_2_.

As mentioned earlier, particle size also plays an important role. Figure 7 presents the graphs of the particle size of the obtained nanocomposites, and the detailed measurement results are presented in Table 3. The average particle size is 100 to 200 nm, which is due to the tendency to form larger agglomerates.

### 3.3. The Influence of the Environment on the Morphology of the Obtained Nanocomposites

#### 3.3.1. Influence of TEOS on Nanocomposite Morphologies

**Figure 8:**
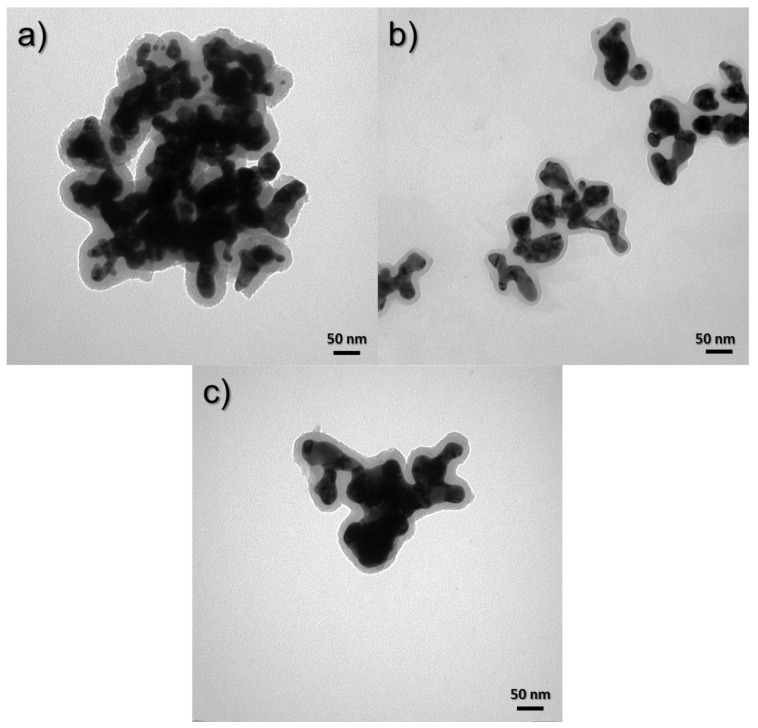
TEM images of syntheses to compare the effect of TEOS concentration: (**a**) 4.38 × 10^−3^ M (1st synthesis), (**b**) 2.8 × 10^−3^ M (2nd synthesis) and (**c**) 2.33 × 10^−3^ M (6th synthesis).

To determine the morphology of the obtained *core–shell* nanostructures, images were taken using a TEM. The images show a clear difference between the core and the shell. The black color indicates the presence of a gold nanoparticle, while the gray coating indicates a layer of silica. Figure 8 shows the effect of silica precursor (TEOS) on the size and shape of nanostructures. The nanostructures were obtained using TEOS concentrations of 4.38 × 10^−3^ (Figure 8a), 2.8 × 10^−3^ (Figure 8b), and 2.33 × 10^−3^ M (Figure 8c). It can be seen that, as the concentration in the sample decreases, the thickness of the silica sheath around the gold core also decreases. The thickness of the silica shell for the structure obtained by Synthesis 1 is about 30 nm, while, for Synthesis 2, it is about 20 nm, and, for Synthesis 6, where the silica concentration is the smallest, it is about 15 nm.

#### 3.3.2. Influence of CTAB on Nanocomposite Morphologies

**Figure 9:**
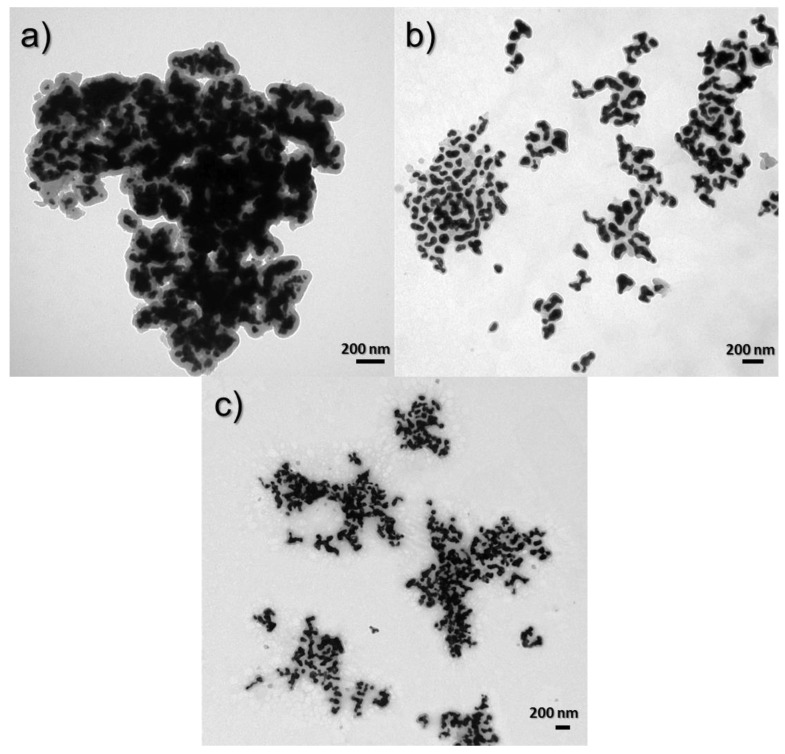
TEM images of syntheses to compare the influence of CTAB concentration: (**a**) 7.47 × 10^−4^ M (Synthesis 1), (**b**) 1.07 × 10^−3^ M (Synthesis 3), and (**c**) 1.6 × 10^−3^ M (Synthesis 5).

Figure 9 shows the TEM images of samples obtained using Syntheses 1, 3, and 5, in which the surfactant concentrations were 7.47 × 10^−4^, 1.07 × 10^−3^, and 1.6 × 10^−3^ M, respectively. In Figure 9a for material obtained from Synthesis 1, where the concentration of CTAB is the lowest, it can be seen that the nanostructures are stuck together and form large clusters of agglomerates. In the next pictures (Figure 9b,c), for materials obtained with the increase in the share of CTAB, it can be seen that the degree of agglomeration decreased because we see here large clusters of smaller nanostructures and they are much more visible as a result.

#### 3.3.3. Influence of Hydrazine on Nanocomposite Morphologies

**Figure 10:**
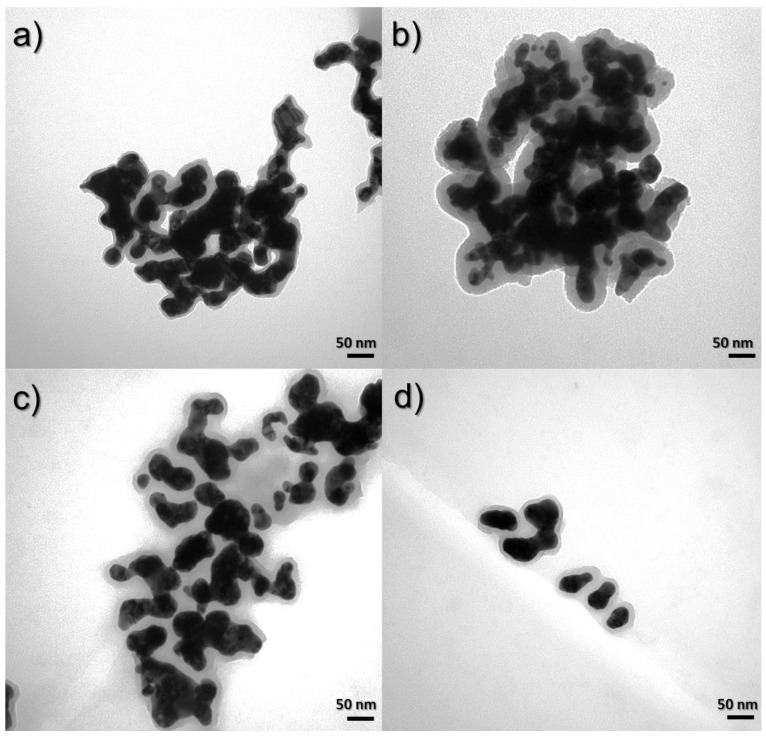
TEM images of syntheses to compare the influence of hydrazine concentration: (**a**) 4.24 × 10^−1^ M (Synthesis 6), (**b**) 3.03 × 10^−1^ M (Synthesis 1), (**c**) 2.43 × 10^−1^ M (Synthesis 3), and (**d**) 1.82 × 10^−1^ M (Synthesis 4).

The effect of reducing the amount of added reducing agent, which is hydrazine, causes the shape of the nanostructures to homogenize. Figure 10 shows the TEM images for the compared samples in this range. In Figure 10a,b, nanomaterials with concentrations of hydrazine in the solution of 4.24 × 10^−1^ and 3.03 × 10^−1^ M are presented. It can be seen that the shape of these nanocomposites is heterogeneous. In addition, images of samples obtained by the third and fourth syntheses, where the concentrations of hydrazine were 2.43 × 10^−1^ and 1.82 × 10^−1^ M, respectively, are presented. In these cases, the shape is more uniform and spherical nanostructures also appear.

### 3.4. Determination of Fmoc Group Loading on Core–Shell Nanostructure

**Figure 11:**
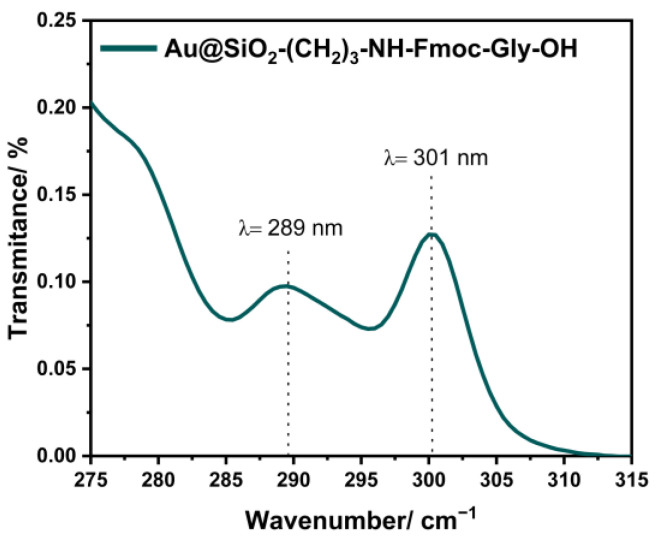
Absorption spectra of Au@SiO_2_–(CH_2_)_3_–NH–Gly–OH.

To determine the deposition number, a spectrophotometric measurement was performed, which allows the concentration of Fmoc groups to be estimated based on the Beer–Lambert law. Moreover, the identification of the number of Fmoc groups allows the subsequent calculation of the amino groups present in the nanostructure before Fmoc–Gly–OH attachment. The determination of the concentration of amino groups bound to Au@SiO_2_ was performed as previously described [25]. In the first stage, the number of Fmoc groups present on the surface of Au@SiO_2_–(CH_2_)_3_–NH_2_ was estimated, which ranges from 15 to 14 µmol/g. Spectrophotometric measurements performed for Au@SiO_2_–(CH_2_)_3_–NH–Gly–Fmoc are shown in Figure 11.

### 3.5. Fluorescence Properties

**Figure 12:**
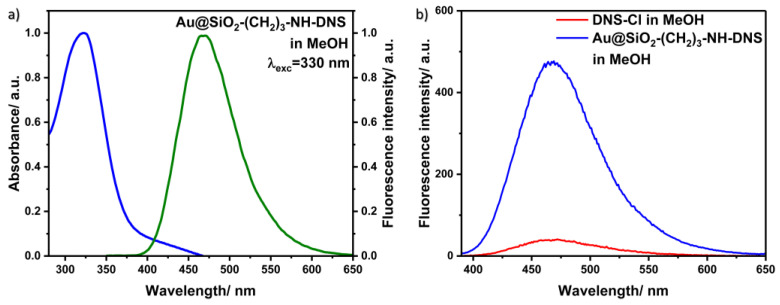
(**a**) Normalized absorption (blue line) and emission spectra (green line) of Au@SiO_2_–(CH_2_)_3_–NH–DNS in methanol solution. (**b**) Fluorescence spectra of Au@SiO_2_–(CH_2_)_3_–NH–DNS and DNS–Cl in methanol.

To search for new functionalized nanomaterials for unique applications, we covalently added DNS–Cl to the core–shell nanostructures obtained in Synthesis 1, which expands the range of possible applications of the nanocomposite. Figure 12a presents the absorption (blue line) and fluorescence spectra (green line) of dansyl covalently bonded to the core–shell Au@SiO_2_ in the MeOH solution. The maximum points of the absorption and fluorescence spectra are located at 323 and 468 nm, respectively. 

On the other hand, Figure 12b shows selected fluorescence spectra of the Au@SiO_2_–(CH_2_)_3_–NH–DNS nanocomposite in the MeOH solution (blue line) and DNS–Cl in methanol solution (red line), with the same concentration as in the above sample for reference. The measurements for these samples were performed under the same conditions. It is known that the plasmonic fluorescence enhancement or quenching strongly depends on the gold–fluorophore distance [34]. In the case of core–shell nanostructures, the distance between gold and fluorophore can be easily tuned by the silica shell thickness used. In our case, the intensity of the fluorescence spectrum of Au@SiO_2_–(CH_2_)_3_–NH–DNS nanocomposite is about 12 times higher than that of DNS–Cl. The fluorescence enhancement effect results from the electromagnetic interaction of light-excited plasmons located on the surface of the metallic core of the tested nanostructures with connected dansyl molecules [3,18].

Coating the core with layers of appropriate thickness of SiO_2_ can improve not only the reactivity of nanostructures, protect against the phenomenon of excessive aggregation, and reduce the cytotoxicity of the synthesized systems, but, from the point of view of amplifying the fluorescence signal, a properly selected SiO_2_ layer also prevents the effect of strong fluorescence quenching, which occurs due to energy transfer through gold surfaces when they are very close to the fluorophores (less than 4 nm) [18,19].

## 4. Conclusions

In summary, we successfully developed the preparation method for structurally stable Au@SiO_2_ *core–shell* nanostructures and furthered their modification with dansyl chloride. First, Au@SiO_2_ nanostructures were characterized by TEM, UV–Vis, and FT-IR. In addition, zeta potential and particle size measurements were performed. The maximum located at around 550 nm confirms the presence of gold. In the FT-IR spectra, bands of stretching vibrations at 1102 cm^−1^ corresponding to Si–O–Si can be seen, which confirm that a nanocomposite structure was created. Zeta potential measurements showed that all samples were stable as each had an average zeta potential greater than +25 mV. And the average size of the nanocomposite was in the range of 100–200 nm. Comparing the influence of reaction conditions on the structure of the obtained nanocomposites, the following conclusions were drawn:

- With increasing concentration of tetraethoxysilane, i.e., the silica precursor, the thickness of the shell increases;
- Increasing the share of CTAB in the synthesis, the degree of agglomeration of Au@SiO_2_ decreases;
- With decreasing concentration of hydrazine, which acts as a reducer in the synthesis, shape and size are more uniform.

In addition, we determined the number of amino group depositions and confirmed the covalent attachment of the DNS fluorophore to Au@SiO_2_ nanostructures through the amino groups. The results showed that, due to tuning of the silica shell thickness, the intensity of the fluorescence spectrum of Au@SiO_2_–(CH_2_)_3_–NH–DNS nanocomposite is about 12 times higher than that of DNS–Cl. The obtained high enhancement is a very promising result for potential applications, e.g., for sensitivity detection of vitamins, oncological drugs, trace amounts of chemical compounds, or activities of enzymes.

## Figures and Tables

**Table 1:** Comparison of the influence of reagents.

		*Influence of the Concentration of a Reagent on the Morphology of the* *Obtained Nanostructures [mol/dm^3^]*		
		TEOS	CTAB	Hydrazine
**Number of Au@SiO_2_ sample**	**1**	4.38 × 10^−3^	7.47 × 10^−4^	3.03 × 10^−1^
	**2**	2.8 × 10^−3^	7.47 × 10^−4^	3.03 × 10^−1^
	**3**	2.8 × 10^−3^	1.10 × 10^−3^	2.43 × 10^−1^
	**4**	2.8 × 10^−3^	1.10 × 10^−3^	1.82 × 10^−1^
	**5**	2.8 × 10^−3^	1.60 × 10^−3^	3.03 × 10^−1^
	**6**	2.33 × 10^−3^	1.10 × 10^−3^	4.24 × 10^−1^

**Table 2:** The mean results of zeta potential.

Number of Au@SiO_2_ Sample	Mean Zeta Potential (SD) [mV]	Deviation [mV]
1	51.8	1.7
2	44.8	2.1
3	49.4	1.6
4	50.7	1.2
5	54.3	2.5
6	45.3	1.0

**Table 3:** Results of the measurement of particle size distribution.

Number of Au@SiO_2_ Sample	Average Size (SD) [nm]	Standard Deviation [nm]	Area [%]
1	196.37	90.95	100.00
2	175.63	82.34	100.00
3	173.75	67.17	95.66
	18.82	3.36	4.34
4	117.66	51.38	90.58
	15.70	3.52	9.42
5	181.55	76.45	100.00
6	175.91	85.40	94.04
	20.33	4.44	5.96

## Data Availability

The data presented in this study are available on request from the corresponding author (due to privacy).

## References

[1] Eustis S., El-Sayed M.A. (2006). Why Gold Nanoparticles Are More Precious than Pretty Gold: Noble Metal Surface Plasmon Resonance and Its Enhancement of the Radiative and Nonradiative Properties of Nanocrystals of Different Shapes. Chem. Soc. Rev..

[2] Wang Y., Yan B., Chen L. (2013). SERS Tags: Novel Optical Nanoprobes for Bioanalysis. Chem. Rev..

[3] Synak A., Grobelna B., Raut S., Bojarski P., Gryczyński I., Karczewski J., Shtoyko T. (2016). Metal Enhanced Fluorescence of Flavin Mononucleotide Using New Plasmonic Platform. Opt. Mater..

[4] Hu X., Zhang Y., Ding T., Liu J., Zhao H. (2020). Multifunctional Gold Nanoparticles: A Novel Nanomaterial for Various Medical Applications and Biological Activities. Front. Bioeng. Biotechnol..

[5] Anik M.I., Mahmud N., Al Masud A., Hasan M. (2022). Gold Nanoparticles (GNPs) in Biomedical and Clinical Applications: A Review. Nano Sel..

[6] Kim J., Hong C.-O., Koo Y., Choi H.-D., Lee K.-W. (2012). Anti-Glycation Effect of Gold Nanoparticles on Collagen. Biol. Pharm. Bull..

[7] Taufikurohmah T., Sanjaya I.G.M., Baktir A., Syahrani A. (2012). Activity Test of Nanogold for Reduction of Free Radicals, a Pre-Assessment Utilization Nanogold in Pharmaceutical as Medicines and Cosmetics. J. Mater. Sci. Eng. B.

[8] Alanazi F.K., Radwan A.A., Alsarra I.A. (2010). Biopharmaceutical Applications of Nanogold. Saudi Pharm. J..

[9] Li X., Hu Z., Ma J., Wang X., Zhang Y., Wang W., Yuan Z. (2018). The Systematic Evaluation of Size-Dependent Toxicity and Multi-Time Biodistribution of Gold Nanoparticles. Colloids Surf. B Biointerfaces.

[10] Bahadur N.M., Watanabe S., Furusawa T., Sato M., Kurayama F., Siddiquey I.A., Kobayashi Y., Suzuki N. (2011). Rapid One-Step Synthesis, Characterization and Functionalization of Silica Coated Gold Nanoparticles. Colloids Surf. A Physicochem. Eng. Asp..

[11] Tengjisi, Liu Y., Zou D., Yang G., Zhao C.-X. (2022). Bioinspired Core-Shell Silica Nanoparticles Monitoring Extra- and Intra-Cellular Drug Release. J. Colloid Interface Sci..

[12] Kalambate P.K., Dhanjai, Huang Z., Li Y., Shen Y., Xie M., Huang Y., Srivastava A.K. (2019). Core@shell Nanomaterials Based Sensing Devices: A Review. TrAC Trends Anal. Chem..

[13] Chaudhary A., Baijnath, Bharadwaj P., Kumar P., Bhaskarwar A. (2023). Sensing Materials: Bimetallics and Metal Mixtures (Core-Shell Microspheres). Encyclopedia of Sensors and Biosensors.

[14] Dembski S., Schneider C., Christ B., Retter M. (2018). Core-Shell Nanoparticles and Their Use for in Vitro and in Vivo Diagnostics. Core-Shell Nanostructures for Drug Delivery and Theranostics.

[15] Szczepańska E., Synak A., Bojarski P., Niedziałkowski P., Wcisło A., Ossowski T., Grobelna B. (2020). Dansyl-Labelled Ag@SiO_2_ Core-Shell Nanostructures—Synthesis, Characterization, and Metal-Enhanced Fluorescence. Materials.

[16] Synak A., Adamska E., Grobelna B., Gondek J., Mońka M., Gryczynski I., Bojarski P. (2018). Photophysical Properties and Detection of Valrubicin on Plasmonic Platforms. Dye. Pigment..

[17] Kang J.S., Piszczek G., Lakowicz J.R. (2002). Enhanced Emission Induced by FRET from a Long-Lifetime, Low Quantum Yield Donor to a Long-Wavelength, High Quantum Yield Acceptor. J. Fluoresc..

[18] Lakowicz J.R. (2006). Instrumentation for Fluorescence Spectroscopy. Principles of Fluorescence Spectroscopy.

[19] Geddes C.D., Cao H., Gryczynski I., Gryczynski Z., Fang J., Lakowicz J.R. (2003). Metal-Enhanced Fluorescence (MEF) Due to Silver Colloids on a Planar Surface:  Potential Applications of Indocyanine Green to in Vivo Imaging. J. Phys. Chem. A.

[20] Anderson S.D., Gwenin V.V., Gwenin C.D. (2019). Magnetic Functionalized Nanoparticles for Biomedical, Drug Delivery and Imaging Applications. Nanoscale Res. Lett..

[21] Mallick S., Singh K.R., Nayak V., Singh J., Singh R.P. (2022). Potentialities of Core@shell Nanomaterials for Biosensor Technologies. Mater. Lett..

[22] Daruich De Souza C., Ribeiro Nogueira B., Rostelato M.E.C.M. (2019). Review of the Methodologies Used in the Synthesis Gold Nanoparticles by Chemical Reduction. J. Alloys Compd..

[23] Ghimire P.P., Jaroniec M. (2021). Renaissance of Stöber Method for Synthesis of Colloidal Particles: New Developments and Opportunities. J. Colloid Interface Sci..

[24] Wang J., Zheng S., Shao Y., Liu J., Xu Z., Zhu D. (2010). Amino-Functionalized Fe(3)O(4)@SiO(2) Core-Shell Magnetic Nanomaterial as a Novel Adsorbent for Aqueous Heavy Metals Removal. J Colloid Interface Sci.

[25] Szczepańska E., Grobelna B., Ryl J., Kulpa A., Ossowski T., Niedziałkowski P. (2020). Efficient Method for the Concentration Determination of Fmoc Groups Incorporated in the Core-Shell Materials by Fmoc–Glycine. Molecules.

[26] Amendola V., Pilot R., Frasconi M., Maragò O.M., Iatì M.A. (2017). Surface Plasmon Resonance in Gold Nanoparticles: A Review. J. Phys. Condens. Matter.

[27] Adamska E., Niska K., Wcisło A., Grobelna B. (2021). Characterization and Cytotoxicity Comparison of Silver- and Silica-Based Nanostructures. Materials.

[28] Montaño-Priede J.L., Coelho J.P., Guerrero-Martínez A., Peña-Rodríguez O., Pal U. (2017). Fabrication of Monodispersed Au@SiO_2_ Nanoparticles with Highly Stable Silica Layers by Ultrasound-Assisted Stöber Method. J. Phys. Chem. C.

[29] Ramalla I., Gupta R., Bansal K. (2015). Effect on Superhydrophobic Surfaces on Electrical Porcelain Insulator, Improved Technique at Polluted Areas for Longer Life and Reliability. Int. J. Eng. Technol..

[30] Liu D., Wang J., Zhang Y., Liu J., Li H., Zhou L., Wu S., Gao X. (2018). Preparation of Core–Shell Structured Au@O_2_ Nanocomposite Catalyst with Au Core Size below 2 Nm without High-Temperature Calcination Procedure. J. Mater. Sci..

[31] Bhattacharjee S. (2016). DLS and Zeta Potential—What They Are and What They Are Not?. J. Control. Release.

[32] Prathna T.C., Chandrasekaran N., Raichur A.M., Mukherjee A. (2011). Kinetic Evolution Studies of Silver Nanoparticles in a Bio-Based Green Synthesis Process. Colloids Surf. A Physicochem. Eng. Asp..

[33] Zhang Z., Guo Z., Yang W. (2022). Cetyltrimethylammonium Bromide Assisted Preparation of Au@O_2_ Particles. Colloid Interface Sci. Commun..

[34] Li J.-F., Li C.-Y., Aroca R.F. (2017). Plasmon-Enhanced Fluorescence Spectroscopy. Chem. Soc. Rev..

